# Design of a Large-Format Scene Simulation System Based on Multi-DMD Optical Stitching

**DOI:** 10.3390/s26041347

**Published:** 2026-02-20

**Authors:** Zheng Liu, Jie Li, Xiayang Huang, Pengxi Liu, Wennan Cui, Tao Zhang

**Affiliations:** 1 School of Information Science and Technology, ShanghaiTech University, Shanghai 201210, China; liuzheng@shanghaitech.edu.cn; 2 Shanghai Institute of Technical Physics, Chinese Academy of Sciences, Shanghai 200083, China; lijie183@mails.ucas.ac.cn (J.L.);; 3 University of Chinese Academy of Sciences, Beijing 100049, China

**Keywords:** digital micromirror device, large-format target plane, scene simulation system, Offner relay system, off-axis three-mirror system

## Abstract

As a widely used display device, the effective display area of a digital micromirror device (DMD) is limited by its micromirror count and pitch, which cannot meet large-format target-plane display requirements. This paper proposes a large-format scene simulation system based on multi-DMD optical stitching, and uses an optical relay to overcome the inability to directly tile DMDs because of their package frames. By constructing a DMD display architecture comprising an illumination module, a relay module, a stitching module, and a projection module, the system quadruples the effective display area relative to a single DMD without sacrificing frame rate. Design results show that the system achieves an MTF greater than 0.5 at 50 lp/mm, with near-diffraction-limited performance; the RMS spot radius is less than 10 μm. All key indicators meet application requirements.

## 1. Introduction

With the rapid development of aerospace technology, photoelectric detection systems for high-speed dynamic targets have put forward higher requirements for the scene generation and ground verification capabilities featuring large area array and high frame rate [1,2,3]. Although field tests yield results with high reliability close to real-world applications, they suffer from high costs, long development cycles, and severe constraints imposed by environmental factors including meteorology, illumination, atmospheric turbulence, ground object backgrounds and platform attitudes. It is thus difficult to cover all working conditions with sufficient traversal, and the repeatability is poor—for instance, the control and allocation of airspace and test ranges often force temporary adjustments to test windows and procedures, making it impossible to maintain consistent working conditions across different test batches. For the above reasons, large-format scene simulation systems, which can simulate dynamic scenes under laboratory conditions to conduct a comprehensive test of various system indicators, are of great significance for shortening the development cycle and reducing experimental risks [4,5,6,7]. In the research, development and testing of scene simulation systems, the most critical and challenging aspect is the realistic reproduction of actual application scenarios. On the one hand, in typical application scenarios such as satellite remote sensing and airborne photoelectric reconnaissance, the pixel scale of large-area array detectors has generally reached 1920×1080 or higher. The large format directly determines the scale and resolution of the simulated field of view, which is a core prerequisite for matching large area array detectors and achieving high-fidelity full-field-of-view testing. On the other hand, detected targets mostly exhibit characteristics of high-speed maneuvering and strong time variation, and the effective temporal frequency of their image features at the pixel scale can reach the hundred-hertz level. According to the Nyquist sampling criterion, to avoid temporal aliasing and accurately reproduce the dynamic characteristics of targets, the effective refresh rate of a large-format scene simulation system must reach at least 200 Hz. In combination with the detector integration time and system timing margin, higher frame rate output is the key to reducing dynamic distortion [8,9,10,11]. These issues together mean that traditional solutions can hardly meet the requirements of system-level functional and performance verification for large area array and high frame rate detectors [12,13,14].

Among the core devices used in scene simulation system, digital micromirror devices (DMDs) stand out for their high frame rate, high contrast, high brightness, and fast response [15]. However, the target-plane size (effective display area) of a single DMD is determined by the micromirror count and pitch and is limited by semiconductor fabrication processes; in the near term, it is difficult to scale significantly. This has become a bottleneck to further improvements in field of view, resolution, and exit pupil. To address this limitation, Texas Instruments introduced the DLP660TE, which is based on a 2716×1528 micromirror array and achieves 4K UHD (3840×2160) display using Expanded Pixel Resolution (XPR) technology [16]. However, the data throughput required by XPR constrain DLP660TE’s maximum input frame rate to 60 Hz, limiting its use in high-frame-rate applications. Hence, it is not suitable where high frame rates are critical. Alternatively, multi-chip DMD stitching can effectively enlarge equivalent target-plane aperture and stop via optical/physical stitching without relying on single-chip process breakthroughs, making it a feasible approach for large-format scene simulation. In prior work, Yu Pengliang et al. from Harbin New Light Optoelectronics proposed a dual-DMD internal beam-combining method for high-resolution midwave infrared (MWIR) imaging simulation [17]. Two 1920×1080 DMDs were used as imaging devices, with a high-temperature blackbody simulating the target, a low-temperature blackbody simulating the background, and Köhler illumination providing uniform lighting. The MWIR beams from the two DMDs were internally combined via a zero-curvature beam-combining element and then projected as collimated output; the projector output was coupled into the tested device’s entrance pupil and imaged onto its detector, achieving a 2K×2K mid-wave infrared target and background simulation. However, that scheme’s effective target-plane expansion was limited by the dual-chip scale and internal beam-combining geometry, and lacked extensibility—making it difficult to meet verification needs for large-format, high-frame-rate applications.

To overcome these issues, this paper proposes a high-frame-rate, large-format scene simulation system based on multi-DMD stitching. A reflective illumination scheme is adopted to uniformly illuminate the DMDs. An optical relay system is used to overcome the inability to directly tile DMDs due to their package frames. Each relay path reflects the DMD output beam, folds it via mirrors toward a spherical mirror, then to a second spherical mirror, and after three reflections forms a relay intermediate image plane. The relay intermediate image planes are stitched using prism-based beam folding, with four DMD channels arranged in rotational symmetry. A projection module then converts the stitched image into a collimated beam, simulating dynamic scenes from infinity. By building a DMD display system comprising illumination, relay, stitching, and projection modules, we quadruple the effective display area of a single DMD without any loss of frame rate.

## 2. Optical System Design

**Figure 1:**
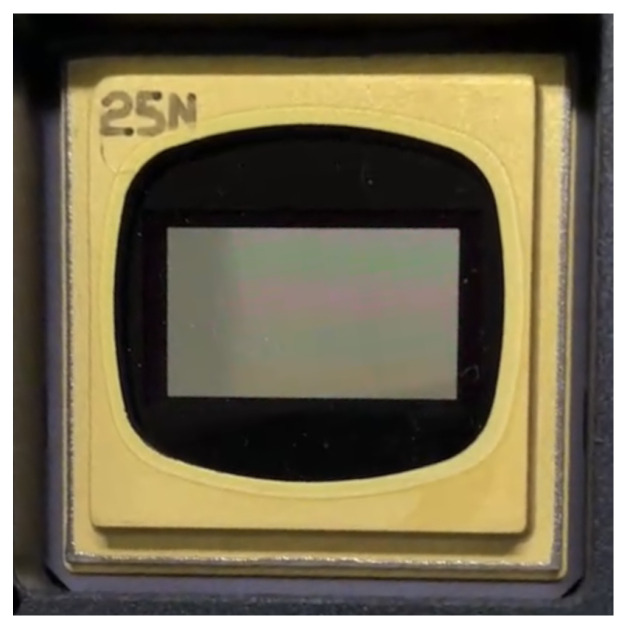
The pictorial diagram of the DLP9500 chipset.

The projector’s optical specifications are determined by the selected DMD and the device under test (DUT). The design adopts the mainstream DMD, the DLP9500 chipset, launched by Texas Instruments (TI). The core technical parameters of this chipset are presented in Table 1 [18]. Figure 1 shows the pictorial diagram of the DLP9500 chipset. As can be seen from the figure, the active imaging area for light modulation in the middle of the chipset is surrounded by a package frame, which serves the functions of structural support and signal extraction. The existence of this package frame makes it impossible for the DLP9500 chipset to achieve seamless stitching of multi-chip imaging areas through direct lamination, thus requiring the design of an additional stitching mechanism.

The large-target-plane scene simulation system studied in this paper provides ground-based HWIL simulation and verification for large-format photoelectric detection equipment. The core interfaces and performance parameters of the device under test (DUT) are listed in Table 2:

Accordingly, the projector must (i) match the spectral band; (ii) provide an exit pupil slightly larger than the DUT entrance pupil to allow alignment margin and avoid vignetting; (iii) slightly exceed the DUT FOV to enable full-FOV simulation; (iv) match the working F-number to ensure radiometric flux and pupil matching; and (v) achieve near-diffraction-limited image quality, i.e., MTF > 0.5 at the Nyquist frequency corresponding to 10 µm pixels (50 lp/mm), and RMS spot radius smaller than one detector pixel.

The operating principle is as follows: white light from a planar source illuminates the DMD surface; the DMD-reflected beam is relayed to a relay intermediate image plane, where multiple sub-images are stitched. The stitched image is then collimated by a three-mirror system to simulate dynamic scenes from infinity. The optical system modeling, image quality evaluation (MTF, spot diagram), tolerance analysis, and Monte Carlo simulation in this study were all performed using Zemax OpticStudio 19.4 (Ansys, Inc., Kirkland, WA, USA) [19]. Consequently, the design must address three aspects:

1. The images from the four DMDs must be seamlessly stitched at the relay intermediate image plane to form a continuous large-format image.
2. All four ray paths (one per DMD) must maintain consistent image quality and resolution after passing through the relay and three-mirror projection systems.
3. The simulator and DUT must satisfy pupil-matching constraints; the simulator’s exit pupil is designed slightly larger than the DUT’s entrance pupil.

Based on this analysis, a reflective optical scheme is adopted. Mirror-based imaging intrinsically avoids chromatic aberration and secondary spectrum, thus supporting broader spectral coverage and facilitating extension to broader spectral bands. It also reduces dependence on material properties and simplifies selection. Moreover, reflective optics permit compact beam folding, shortening structural length and enabling lightweight design.

### 2.1. Illumination System Design

A reflective, 1:1 imaging illuminator is used to provide uniform illumination at the DMD plane. Spherical mirrors exhibit spherical aberration: for collimated input, marginal and paraxial rays do not focus to the same point, causing blur. To suppress spherical aberration, we introduce aspheric mirrors. The first reflective element is designed as an even aspheric surfaces to reduce spherical aberration and improve beam focusing; the second reflective element is also aspheric, working in concert with the first to further converge the beam and ensure uniform spot intensity.

The surface equation of the aspheric mirror is the core of the design. The general form of an even-order aspheric surface is set as Equation (1):

(1) $$z=\frac{cr^{2}}{1+\sqrt{1-\left(1+k\right)c^{2}r^{2}}}+\sum_{n=2}^{\infty }\alpha_{n}r^{2n}.$$

where *z* is the surface height along the optical axis direction; *c* is the curvature of the surface vertex ($c=\frac{1}{R}$, where *R* is the radius of curvature); *k* is the conic coefficient; $a_{n}$ is the even-order term coefficient; *r* is the radial coordinate ($r=\sqrt{x^{2}+y^{2}}$).

**Figure 2:**
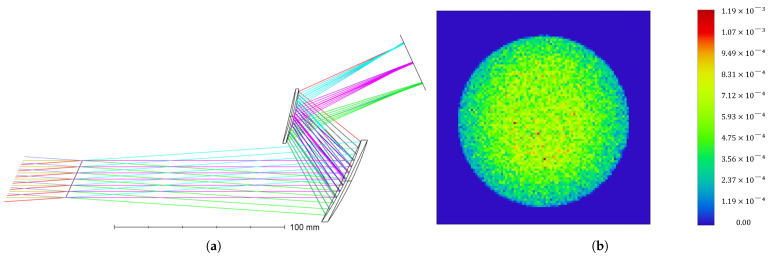
Optical path of the illumination system and simulated uniformity of the illumination spot on the DMD. (**a**) Schematic diagram of the illumination system. (**b**) Pseudocolor energy distribution of the illumination spot over the DMD.

Using these definitions, the initial structure is established and optimized under constraints on effective focal length (EFFL) and working F-number (WFNO). The final design delivers a uniform illumination spot at the DMD. The illumination system parameters are summarized in Table 3, and the ray path is shown schematically in Figure 2a. To verify the uniformity of the illumination spot, geometric optics simulation analysis was performed on the image plane of the illumination system using Zemax software, and the pseudocolor distribution result of the illumination spot is shown in Figure 2b. It can be seen from the simulation results that the spot energy distribution within the effective working area of the DMD is uniform, the spot size consistency is excellent, and there is no obvious brightness difference. The light transmittance efficiency of the system reaches 84.633%, which demonstrates that the designed illumination system can achieve high-uniformity illumination on the DMD surface.

To suppress the noise introduced by multiple reflections of stray light when the DMD is in the off state and improve the signal-to-noise ratio of the system, a stray light absorption structure is integrally designed in the illumination module of this system: a reflector mirror is adopted to directionally guide the reflected light deviated from the main optical path by the DMD in the off state to a high-absorptivity area inside the cavity for source absorption, fundamentally reducing the generation and propagation of stray light. Meanwhile, the inner walls of all optical cavities of the illumination module, relay module and projection module are roughened via anodic oxidation blackening process. By increasing the diffuse reflection loss of stray light on the inner cavity walls, the continuous absorption and attenuation of stray light undergoing multiple reflections inside the cavities are achieved.

### 2.2. Relay System Design

Because the DMD’s active area is surrounded by a package frame, DMDs cannot be directly stitched. An optical relay is required to re-image the DMD active area with unit magnification (1:1) for subsequent stitching. The Offner relay system is a concentric three-reflection optical system. Concentric systems under telecentric conditions automatically correct third-order aberrations and offer excellent imaging performance, while being simple, lightweight, easy to assemble, convenient to fabricate, and cost-effective [20]. Therefore, an Offner relay system is adopted here.

**Figure 3:**
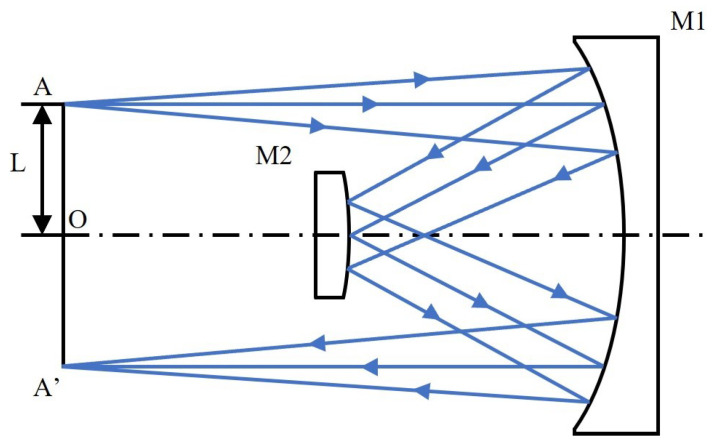
Schematic diagram of the typical Offner system.

As shown in Figure 3, a typical Offner system comprises two concentric spherical mirrors, a concave primary mirror M1 and a convex secondary mirror M2, with radii $R_{1}$ and $R_{2}$, respectively, and a common center *O* on the optical axis. Rays from object point *A* first reflect off one aperture region of M1, then off M2, then a second time off the opposite aperture region of M1, and finally form image $A^{\prime }$ on the image side. In the standard 1:1 Offner relay, *A* and $A^{\prime }$ are axially symmetric about *O* and lie in the same plane. When the aperture stop is located at M2, the system exhibits a small annular field of view. The distance *L* from *A* to *O* is given by Equation (2):

(2) $$L=\frac{R_{1}-\sqrt{4R_{2}^{2}-R_{1}^{2}}}{2}.$$

*H* is defined as the ratio of the field of view radius *L* to $R_{2}$. According to the flat image field condition of the three-reflection system, $R_{1}=2R_{2}$ is taken here, so the formula can be simplified as Equation (3):

(3) $$H=\frac{L}{R_{2}}=\frac{R_{1}-\sqrt{4R_{2}^{2}-R_{1}^{2}}}{2R_{2}}.$$

Within the annular field of view, the chief rays in the object and image spaces remain parallel to each other and perpendicular to the object and image planes, respectively, satisfying the ideal imaging constraints of a concentric reflective system. Since the light beam undergoes two reflections on the primary mirror and an additional reflection on the secondary mirror, the Offner configuration is inherently a three-reflection concentric system. This strictly axisymmetric geometry enables mutual cancellation of third-order aberrations (such as coma, astigmatism, and distortion) under 1:1 relay imaging conditions, resulting in excellent imaging quality while simultaneously exhibiting double-telecentric optical characteristics in both object and image spaces.

**Figure 4:**
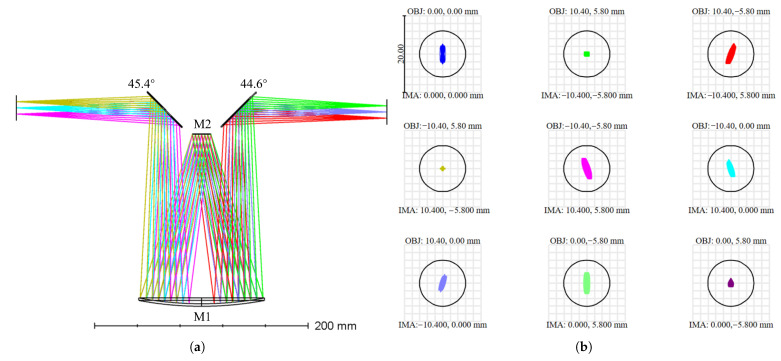
Optical design and imaging performance of the relay system. (**a**) Schematic diagram of the relay system. (**b**) RMS spot diagram of full field of view.

Subject to stitching and mechanical clearance constraints, two plane mirrors are added to the traditional Offner optical system here. The two plane mirrors are arranged near the secondary mirror and mounted on the same structural component as the secondary mirror, which can not only preserve relative alignment but also facilitate subsequent alignment. The reflectors are arranged near the pupil, and their main functions are to adjust the direction of the chief ray and the attitude of the image space coordinate system. By enabling compact beam folding and ensuring mechanical clearance, these two plane mirrors introduce negligible additional aberrations. The fine adjustment of focal-plane tilt required by the system is realized by this pair of reflectors: the angles between the upper and lower reflectors and the optical axis are designed to be 44.6° and 45.4° respectively. While maintaining a deflection close to 90°, a slight rotation of the image-space coordinate system is introduced to meet stitching and vignetting requirements. Thus, without sacrificing the image quality, the DMD working surface is kept parallel to the system optical axis and meets the interface and assembly constraints. The parameters of the relay system obtained through final constraint optimization are shown in Table 4, and the ray path diagram of the relay system is shown in Figure 4.

### 2.3. Stitching System Design

**Figure 5:**
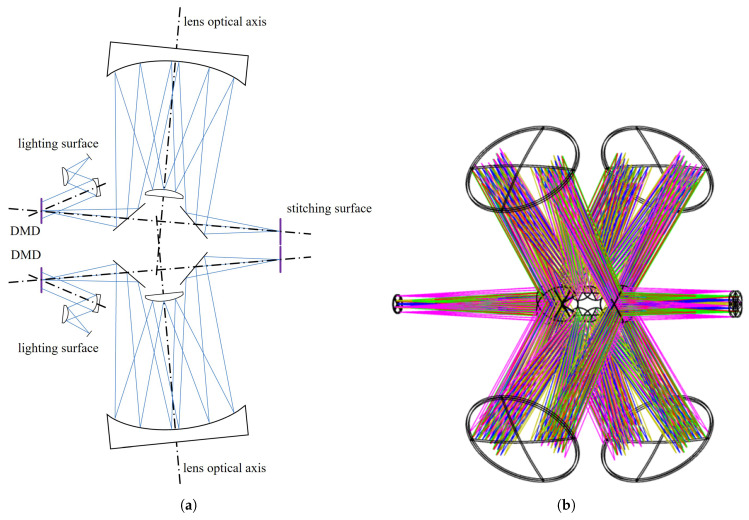
Schematic diagram of the stitching system. (**a**) Schematic diagram of the stitching optical path. (**b**) Schematic diagram of 3D stitching.

To avoid mechanical interference among four DMD relays, we adopt a compact, staggered layout that brings their intermediate images to a common stitching plane. The overall stitching ray path is illustrated in Figure 5. This approach minimizes energy loss at the stitching seams. Moreover, the projection system’s image-side numerical aperture is kept modest, supporting the overall system performance.

To avoid spatial structural interference and reduce the structural length, a prism deflection scheme is adopted at the primary image plane to achieve the spatial stitching of four relay intermediate image planes. BK7 optical glass is selected as the substrate material for the stitching prisms; its stable mechanical properties and machinability can guarantee the structural precision of the 45° core refracting surface, while providing a reliable bearing substrate for the reflective function. A high-reflectivity silver-based coating is applied to the reflective surfaces, which reduces the constraints caused by angular dependence compared with total internal reflection (TIR) and improves the tolerance of the system to assembly errors. The BK7 prisms are only used as a high-precision mechanical positioning and assembly reference carrier for the silver-based reflective coatings, rather than transmissive optical elements. The light beam does not propagate through the interior of the prism glass and has no contact with the air–glass transmission interface. It only realizes the optical path deflection and multi-channel beam combining through the 45° silver-based high-reflection coating surface on the prism surface, which is consistent with the optical principle of the independent plane reflector and fundamentally avoids the Fresnel transmission loss caused by the prism structure.

**Figure 6:**
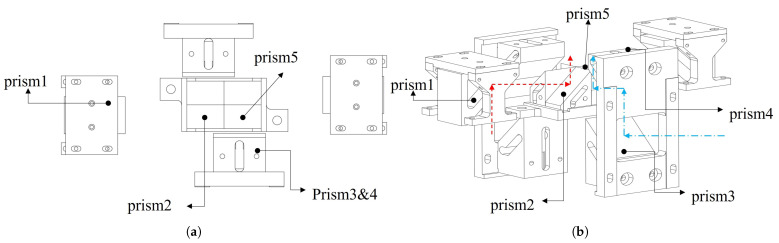
Schematic of the stitching prism. (**a**) Prism assembly configuration. (**b**) Beam steering paths: red arrows show the two-reflection beam path through Prism 1 and Prism 2; blue arrows show the three-reflection beam path through Prism 3, Prism 4 and Prism 5.

As shown in Figure 6a, the four central prisms are arranged in C4 rotational symmetry, among which Prism 2 and Prism 5 serve as the core central prisms and are fixed to the base plate via an integral bonding process. After the four central prisms are bonded to the base plate as a whole, they are firmly mounted on the structural substrate by a pressing plate assembly to ensure the precision of the symmetrical layout. Prism 1, Prism 3 and Prism 4 are dedicated prisms for deflected optical paths and are fixed by a composite method of “bonding + screw connection”: the prisms are first precisely bonded to the structural base plate, and then detachably connected to the structural substrate by screws. This design facilitates fine adjustment of the prism attitude during assembly to ensure that the light beams propagate strictly along the predetermined paths. There are two main equivalent forms of the deflected optical paths: as indicated by the red arrows in Figure 6b, the light beams pass through Prism 1 and Prism 2 in sequence and reach the stitched image plane after two reflections; as indicated by the blue arrows in Figure 6b, the light beams pass through Prism 3, Prism 4 and Prism 5 in sequence and reach the stitched image plane after three reflections.

Because the reflection count differs between channels, image flip inversion (mirror reversal) may occur. We pre-compensate this via a pixel-space mapping at the DMD input, ensuring consistent orientation across all four quadrants. This precisely cancels the image-space transformation induced by differing reflection counts, without adding extra reflective elements, thereby avoiding additional energy loss.

### 2.4. Projection System Design

**Figure 7:**
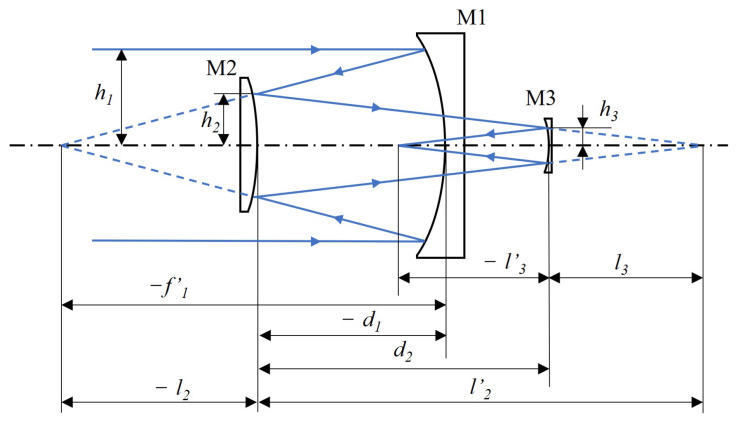
Schematic of the stitched ray path.

The projection system is designed based on the data listed in Table 2 and the corresponding requirements for the projection system. Here, an off-axis three-mirror system is used to project the stitched intermediate image plane. The initial scheme of the off-axis reflective optical system usually first obtains the first-order solution based on the coaxial three-mirror structure, and then realizes the goal of no obstruction in the central light-transmitting area through off-axis cropping [21]. For the calculation of the coaxial initial structure (see Figure 7), it is assumed that the primary mirror M1, the secondary mirror M2, and the tertiary mirror M3 all adopt aspheric surfaces, and the object side is at infinity. Therefore, the object distance in front of the first surface is taken as $l_{1}=\infty$, and the inclination angle of the chief ray with the system optical axis is taken as $u_{1}=0$. Define the geometric obstruction ratio of the secondary mirror relative to the primary mirror as $\alpha_{1}$, and the geometric obstruction ratio of the tertiary mirror relative to the secondary mirror as $\alpha_{2}$; at the same time, let the linear magnifications of the secondary mirror and the tertiary mirror be $\beta_{1}$ and $\beta_{2}$ respectively. Under these settings, the first-order constraints can be expressed as Equation (4):

(4) $$\begin{array}{cccc} \alpha_{1} & =\frac{l_{2}}{f_{1}^{\prime }}\approx \frac{h_{2}}{h_{1}}, & \alpha_{2} & =\frac{l_{3}}{l_{2}^{\prime }}\approx \frac{h_{3}}{h_{2}},\\ \beta_{1} & =\frac{l_{2}^{\prime }}{l_{2}}=\frac{u_{2}}{u_{2}^{\prime }}, & \beta_{2} & =\frac{l_{3}^{\prime }}{l_{3}}=\frac{u_{3}}{u_{3}^{\prime }}. \end{array}$$

Considering the influence of the quadric aspherical surface on the system aberration, the calculation formulas of the corresponding third-order aberration coefficients are as follows [22]:

(5) $$\begin{array}{cc} S_{I} & =\frac{1}{4}[\left(e_{1}^{2}-1\right)\beta_{1}^{3}\beta_{2}^{3}-e_{2}^{2}\alpha_{1}\beta_{2}^{3}{\left(1+\beta_{1}\right)}^{3}\\ \; & \;+e_{3}^{3}\alpha_{1}\alpha_{2}{\left(1+\beta_{2}\right)}^{3}+\alpha_{1}\beta_{2}^{3}\left(1+\beta \right){\left(1-\beta_{1}\right)}^{2}\\ \; & \;-\alpha_{1}\alpha_{2}\left(1+\beta_{2}\right){\left(1-\beta_{2}\right)}^{2}], \end{array}$$

(6) $$\begin{array}{cc} S_{\mathrm{II}} & =-\frac{e_{2}^{2}\left(\alpha_{1}-1\right)\beta_{2}^{3}{\left(1+\beta_{1}\right)}^{3}}{4\beta_{1}\beta_{2}}\\ \; & \;+\frac{e_{3}^{2}\left[\alpha_{2}\left(\alpha_{1}-1\right)+\beta_{1}\left(1-\alpha_{2}\right)\right]{\left(1+\beta_{2}\right)}^{2}}{4\beta_{1}\beta_{2}}\\ \; & \;+\frac{\left(\alpha_{1}-1\right)\beta_{2}^{3}\left(1+\beta_{1}\right){\left(1-\beta_{1}\right)}^{2}}{4\beta_{1}\beta_{2}}\\ \; & \;-\frac{\left[\alpha_{2}\left(\alpha_{1}-1\right)+\beta_{1}\left(1-\alpha_{2}\right)\right]\left(1+\beta_{2}\right){\left(1-\beta_{2}\right)}^{2}}{4\beta_{1}\beta_{2}}-\frac{1}{2}, \end{array}$$

(7) $$\begin{array}{cc} S_{\mathrm{III}} & =-e_{2}^{2}\frac{\beta_{2}{\left(\alpha_{1}-1\right)}^{2}{\left(1-\beta_{1}\right)}^{2}}{4\alpha_{1}\beta_{1}^{2}}\\ \; & \;+e_{3}^{2}\frac{{\left[\alpha_{2}\left(\alpha_{1}-1\right)+\beta_{1}\left(1-\alpha_{2}\right)\right]}^{2}{\left(1+\beta_{2}\right)}^{3}}{4\alpha_{1}\alpha_{2}\beta_{1}^{2}\beta_{2}^{2}}\\ \; & \;+\frac{\beta_{2}{\left(\alpha_{1}-1\right)}^{2}\left(1+\beta_{1}\right){\left(1-\beta_{1}\right)}^{2}}{4\alpha_{1}\beta_{1}^{2}}\\ \; & \;-\frac{{\left[\alpha_{2}\left(\alpha_{1}-1\right)+\beta_{1}\left(1-\alpha_{2}\right)\right]}^{2}\left(1+\beta_{2}\right){\left(1-\beta_{2}\right)}^{2}}{4\alpha_{1}\alpha_{2}\beta_{1}^{2}\beta_{2}^{2}}\\ \; & \;-\frac{\beta_{2}\left(\alpha_{1}-1\right)\left(1-\beta_{1}\right)\left(1+\beta_{1}\right)}{\alpha_{1}\beta_{1}}\\ \; & \;-\frac{\left[\alpha_{2}\left(\alpha_{1}-1\right)+\beta_{1}\left(1-\alpha_{2}\right)\right]\left(1-\beta_{2}\right)\left(1+\beta_{2}\right)}{\alpha_{1}\alpha_{2}\beta_{1}\beta_{2}}\\ \; & \;-\beta_{1}\beta_{2}+\frac{\beta_{2}\left(1+\beta_{1}\right)}{\alpha_{1}}-\frac{1+\beta_{2}}{\alpha_{1}\alpha_{2}}, \end{array}$$

(8) $$\begin{array}{cc} S_{\mathrm{IV}} & =\beta_{1}\beta_{2}-\frac{\beta_{2}\left(1+\beta_{1}\right)}{\alpha_{1}}+\frac{1+\beta_{2}}{\alpha_{1}\alpha_{2}}. \end{array}$$

Among them, $S_{I-\mathrm{IV}}$ are spherical aberration, coma, astigmatism, and field curvature respectively, and $e_{1}^{2}$, $e_{2}^{2}$ and $e_{3}^{2}$ are the quadric surface coefficients of the primary mirror, secondary mirror, and tertiary mirror respectively. By setting $S_{I}=S_{\mathrm{II}}=S_{\mathrm{III}}=S_{\mathrm{IV}}=0$ and performing an aberration-free solution, the initial structural parameters and quadric surface coefficients of each reflecting surface of the three-mirror system can be obtained.

For a parallel light output system, the image height *y*, field angle ω, and focal length *f* satisfy:

(9) y=f·tanω

In addition, the F-number, focal length *f*, and exit pupil diameter *D* satisfy:

(10) $$D=\frac{f}{F}$$

**Figure 8:**
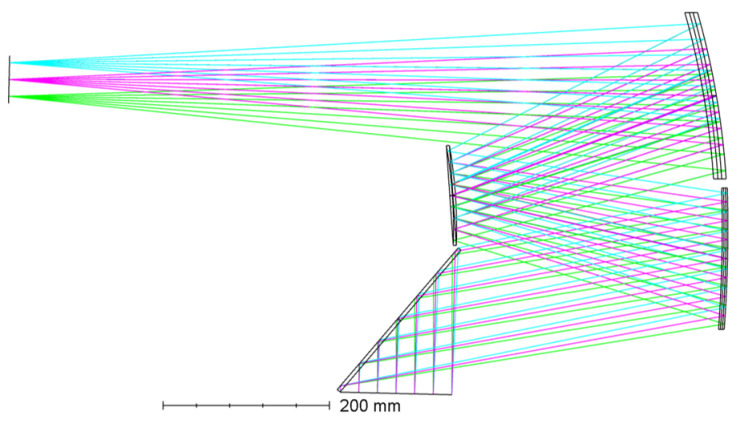
Schematic diagram of the off-axis three-mirror system.

Plane mirrors are introduced after multi-parameter optimization to fold the beam and significantly shorten the axial length. After optimization converges, the key parameters of the three-mirror system are summarized in Table 5 and Table 6, with a schematic ray path in Figure 8. The back focal distance and the spacing between the exit pupil and the secondary mirror provide sufficient margin for placing plane fold mirrors and satisfy alignment and assembly tolerances.

**Figure 9:**
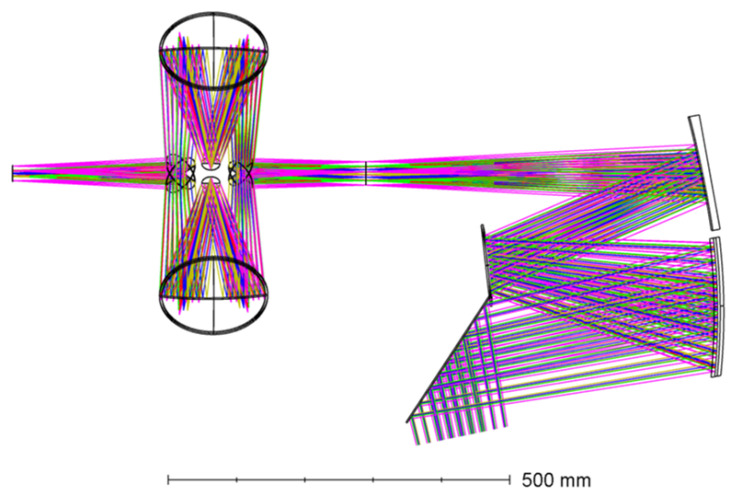
Schematic diagram of the multi-DMD scene simulation system.

For a more intuitive demonstration of the proposed scheme, the complete optical path diagram of the multi-DMD scene simulation system is presented in Figure 9, which serves as the basis for the subsequent optical system performance evaluation.

## 3. Optical System Performance Evaluation

### 3.1. Image Quality Evaluation

Modulation transfer function (MTF) is a primary measure of optical system performance. The designed system is evaluated using MTF and spot diagrams.

The Nyquist frequency $f_{N}$ of the detector is given by

(11) $$f_{N}=\frac{1}{2p}$$

where p=10μm is the pixel pitch. Substituting the value yields a reference spatial frequency of 50 lp/mm.

**Figure 10:**
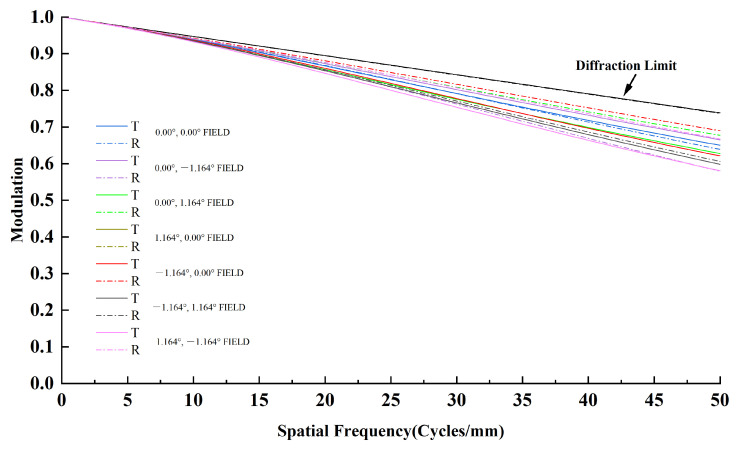
System MTF diagram.

As shown in Figure 10, the system achieves MTF greater than 0.5 at 50 lp/mm, meeting the design requirement.

**Figure 11:**
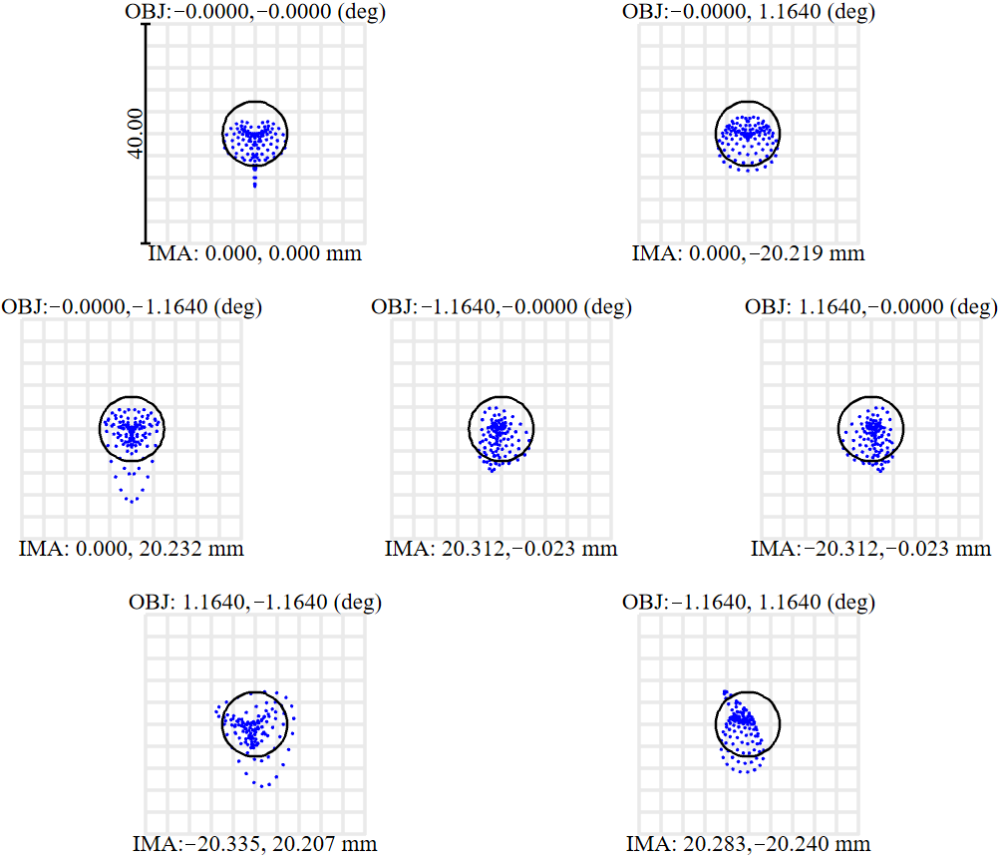
System spot diagram.

The RMS spot radius effectively characterizes energy concentration within the spot. As shown in Figure 11 and summarized in Table 7, the Airy-disk radius is 4.893 μm, while the maximum RMS spot radius across all fields is 4.484 μm—smaller than the Airy-disk radius and less than one detector pixel (10 μm)—indicating excellent imaging quality for the projection system.

### 3.2. Tolerance Analysis

System feasibility depends strongly on tolerance design. Overly tight tolerances render manufacturing and assembly difficult and costly, jeopardizing practical implementation and stability; overly loose tolerances degrade image quality. Therefore, a systematic tolerance analysis is conducted to reasonably allocate margins among design residuals, manufacturing errors, and alignment errors, achieving an optimal balance of performance and cost [23].

Tolerance analysis is performed in Zemax. Error sources are grouped by manufacturability and alignment characteristics, and then random sampling is applied to them. The diffraction MTF at 50 lp/mm is used as the merit function, and 200 Monte Carlo runs are executed. The back focal distance is selected as a compensator, and default tolerance are adjusted to reflect realistic manufacturing and alignment conditions. The final tolerance allocation scheme is listed in Table 8, showing all tolerances within practical manufacturing and assembly capability. Monte Carlo statistics are summarized in Table 9.

### 3.3. Discussion

This work addresses the stringent challenges posed by large-format, high-frame-rate optoelectronic detection systems in ground scene-simulation verification—particularly the bottleneck imposed by the limited effective display area of single DMD—by proposing and successfully designing a multi-DMD optical stitching architecture for large-format scene simulation. The system adopts a reflective optical scheme and integrates illumination, relay, stitching, and projection modules to quadruple the effective display area of a single DMD without any loss of frame rate. In the proposed scheme, all four DMDs are driven in parallel with hardware synchronization, so that the stitched output frame rate equals the signal DMD refresh rate (limited by the DLP9500 controller and data interface).

A core innovation is the modified Offner relay system, which elegantly resolves spatial interference imposed by the DMD package frame and achieves high-quality 1:1 re-imaging of the DMD’s active area. The prism-based folding and spatial stitching strategy accomplishes seamless stitching of the four relayed sub-images, forming a large-format primary image compatible with large-area detectors. Finally, an off-axis three-mirror projection system collimates the stitched image to emulate dynamic scenes from infinity. The design fully considers image-quality consistency across four optical channels and pupil matching. Through rigorous optical design and optimization, the system demonstrates outstanding overall performance.

Notably, the entire optical system of the proposed scheme adopts an all-reflective optical architecture except for the inherent package glass of the DMD device. The illumination module, Offner relay module, prism stitching module and off-axis three-mirror projection module all realize light beam transmission, image transfer and optical path deflection through reflective optical elements, without any air–glass transmission interface in the whole optical path, thus avoiding the generation and accumulation of Fresnel transmission loss. The main light energy loss of the system comes from three aspects: the cumulative loss of film reflectivity caused by multiple reflections (the silver-based high-reflection coating in the visible band of 400–700 nm has high reflectivity, and the total loss is determined by the number of reflections in the optical path), the inherent efficiency loss of the DMD device, which is measured to be approximately 68% under visible illumination, mainly contributed by the diffraction effect, filling factor and micromirror mechanical inclination, as well as the slight light energy loss caused by local optical vignetting and stray light suppression structure [24]. The latter part of the loss has been optimized by precise optical path simulation layout and anodic oxidation blackening treatment of the inner wall of the optical cavity, and the loss ratio is controllable without significantly affecting the overall optical efficiency and imaging quality of the system.

To quantitatively evaluate the optical throughput, the system-level optical efficiency is modeled as a serial product of the efficiencies of each dominant stage, i.e.,

(12) $$\eta_{total}=\prod_{i=1}^{n}\eta_{i}$$

where $\eta_{total}$ is the overall optical efficiency from the illumination output to the projected image plane, and $\eta_{i}$ denotes the efficiency of the i-th stage.

In this design, the key factors are (1) illumination transmission efficiency from the source exit to the DMD plane obtained from Zemax, $\eta_{1}=0.84633$ and (2) cumulative reflectivity loss due to multiple reflections. The silver-based high-reflection coating exhibits a single-bounce reflectance of R=0.98 averaged over 400–700 nm, and the optical train involves N=14 reflective bounces, yielding

(13) $$\eta_{2}=R^{N}=0.98^{14}\approx 0.754$$

(3) the measured intrinsic optical efficiency of the DLP9500 under visible illumination, $\eta_{3}=0.68$, mainly limited by diffraction, fill factor, and micromirror tilt and (4) a minor controllable loss associated with local vignetting and stray-light suppression, conservatively taken as $\eta_{4}=0.95$. Therefore,

(14) $$\eta_{total}=\eta_{1}\eta_{2}\eta_{3}\eta_{4}=0.84633\times 0.754\times 0.68\times 0.95\approx 0.412$$

Indicating an overall optical efficiency of approximately 41.2% and a total optical loss is 58.8%.

It is noteworthy that this reflective optical scheme inherently features low chromatic dispersion and reduced dependence on material properties, enabling the system to be flexibly extended to multiple spectral bands including visible light, short-wave infrared and mid-wave infrared, thus meeting various application requirements in multispectral scene simulation. To further highlight the technical advantages of the proposed architecture, quantitative comparison data are presented in Table 10, which systematically compares the key performance indicators—including effective display area (in comparison with a single DMD), maximum frame rate, resolution and scalability—with the corresponding indicators of existing representative systems.

The proposed large-format scene-simulation architecture overcomes the single-DMD area limitation and provides critical technical support for realistic, efficient laboratory verification of large-format, high-frame-rate optoelectronic systems, with significant engineering value. Future work will focus on further improving energy uniformity across stitching seams, suppressing temporal artifacts under high frame rates, and extending its operation to broader spectral bands (e.g., mid-infrared).

## 4. Conclusions

To meet large-format display requirements in scene simulation, this paper presents a multi-DMD stitching design for a large-format scene simulation system. The reflective optical scheme, combined with an optical relay, resolves the DMD package-frame constraint that prevents direct stitching. The architecture offers advantages including achromatism, compactness, high image quality and high spatial resolution. At the Nyquist frequency of 50 lp/mm, the MTF exceeds 0.5; the maximum RMS spot radius is 4.484 μm. The simulated images exhibit excellent resolution, contrast, and stability, and are suitable for hardware-in-the-loop (HWIL) scene-simulation systems.

## Figures and Tables

**Table 1:** DLP9500 parameters.

Parameter	Value
Illumination wavelength/nm	400–700
Micromirror array size	1920×1080
Micromirror pitch/μm	10.8
Array diagonal/in	0.95
Operating temperature range/°C	20–70
Micromirror array orientation	Orthogonal

**Table 2:** The DUT performance parameters.

Parameter	Value
Spectral Range/nm	400–700
Pixel Size/μm	10
Entrance Pupil Diameter/mm	125
Working F-number	8
Maximum Field of View/°	1.1
Focal Length/mm	1000

**Table 3:** The illumination system parameters.

Parameter	Value
Image Plane Tilt/°	24
Field of View/°	3.86×27.86
Working F-number	3.96

**Table 4:** The key parameters of the relay system.

Parameter	Value
Radius of curvature of M1/mm	319.93
Radius of curvature of M2/mm	161.36
Thickness of M1/mm	158.500
Thickness of M2/mm	−158.500
Clear Semi-Diameter of M1/mm	58.905
Clear Semi-Diameter of M2/mm	8.645
Field of View/°	5.80×10.40
Working F-number	9.34

**Table 5:** The key parameters of the three-mirror system.

Surface	Radius/mm	Conic	Thickness/mm	2nd-Order Aspheric Term
Primary	−1694.990	−1.162	−329.956	−1.097 × 10^−5^
Secondary	−576.848	−0.451	329.706	3.323 × 10^−6^
Tertiary	−941.676	−0.245	−864.133	2.870 × 10^−6^

**Table 6:** The final performance parameters of projection system.

Parameter	Value
Spectral Range/nm	400–700
Effective Focal Length/mm	994.19
Working F-number	7.4
Effective Maximum Field of View/°	1.164
Exit Pupil Diameter/mm	134.34

**Table 7:** RMS spot radius of each field of view (μm).

X-Direction Field of View	Y-Direction Field of View		
	${-1.164}^{{}^{\circ}}$	$0^{{}^{\circ}}$	$1.164^{{}^{\circ}}$
${-1.164}^{{}^{\circ}}$	-	3.620	3.919
$0^{{}^{\circ}}$	4.206	3.852	3.470
$1.164^{{}^{\circ}}$	4.484	3.620	-

**Table 8:** The final tolerance allocation scheme.

Component	Tolerance Parameters						
	Radius/mm	Conic	Tilt X/”	Tilt Y/”	Dec X/mm	Dec Y/mm	Spacing/mm
Primary	±0.5	±0.005	20	20	±0.1	±0.1	±0.1
Secondary	±0.5	±0.001	20	20	±0.1	±0.1	±0.1
Tertiary	±0.4	±0.0004	20	20	±0.1	±0.1	±0.1
Plane Mirror	-	-	20	20	±0.1	±0.1	±0.1

**Table 9:** Monte Carlo analysis results.

Monte Carlo Percentage/%	MTF@50 lp/mm
90	0.51
80	0.54
50	0.59
20	0.62
10	0.63

**Table 10:** Quantitative comparison between the proposed scheme and existing technologies.

Performance Metrics	Proposed Scheme	XPR Technology [16]	Dual-DMD Internal Stitching [17]
Effective Display Area (vs. Single DMD)	4×	1× (4K UHD achieved via XPR) *	2×
Maximum Frame Rate	Consistent with single DMD	60 Hz (limited by data throughput)	Consistent with single DMD
Resolution	3840×2160 (stitched physical pixels)	3840×2160 (equivalent)	1920×2160
Scalability	Support multiple bands (visible light, short wave, medium wave)	Visible light	Medium wave

Data for existing technologies are cited from the corresponding references. * The ”Effective Display Area (vs. Single DMD)” of DLP660TE is recorded as 1× because its 4K UHD resolution is achieved via pixel expansion technology rather than physical or optical stitching, and the actual effective display area is consistent with a single DMD chipset.

## Data Availability

The data included in this experiment are not yet publicly available but can be obtained from the author upon reasonable request.
